# Assessing zoonotic risk in a fenced natural park in northwestern Italy: integrating camera traps for a vector-host approach to investigate tick-borne pathogens

**DOI:** 10.3389/fvets.2025.1536260

**Published:** 2025-03-03

**Authors:** Rachele Vada, Stefania Zanet, Flavia Occhibove, Anna Trisciuoglio, Amir Reza Varzandi, Ezio Ferroglio

**Affiliations:** Department of Veterinary Sciences, University of Turin, Turin, Italy

**Keywords:** humans, Ixodidae, recreational areas, tick-borne zoonoses, wildlife

## Abstract

Tick-borne diseases are among the major widespread emerging zoonotic diseases, and their circulation in the environment is influenced by a broad range of abiotic and biotic factors, including the abundance of vectors and vertebrate hosts. In this study, we estimated the prevalence of tick-borne pathogens and the impact of wildlife head count on their circulation in a lowland natural area in northwestern Italy. We collected ticks and camera trap pictures from 14 sampling points every 2 weeks for 1 year and identified pathogens through molecular analyses: *Babesia capreoli*, *B. microti*-like, *Borrelia burgdorferi sensu lato* (s.l.), *Rickettsia* of the spotted fever group (SFG), *Theileria capreoli*, and *Anaplasma phagocytophilum*. We modeled the presence of *B. capreoli*, *B. microti*-like, *B. burgdorferi* s.l., and SFG *Rickettsia* on head counts of wild ungulates and mesocarnivores. We tested a global model including all collected ticks, as well as a model focusing solely on Ixodes ricinus nymphs, the species, and the developmental stage most associated with zoonotic infection risk. The highest prevalence was obtained for *B. microti*-like (13%) and SFG Rickettsia (11%), and, for most pathogens, no differences were detected among tick species and their developmental stages. Mesocarnivores showed an additive effect on *B. microti*-like and *B. burgdorferi* s.l., while wild ungulates, non-competent for transmission of our target pathogens, showed a dilutive effect. These findings confirm the circulation of relevant tick-borne pathogens in the study area and show the use of camera trap data in predicting tick-borne pathogens’ risk by targeting host species which may have an indirect impact and are more easily addressed by monitoring and control strategies.

## 1 Introduction

In recent years, tick-borne zoonoses have emerged as significant threats to human health, exhibiting increasing prevalence alongside the geographical expansion of their vectors (1–3). Wildlife species can increase the circulation of these pathogens, serving as both reservoirs and hosts for the vectors. However, scant information exists regarding potential differences in pathogen reservoir competence of various species, which is commonly investigated through xenodiagnosis. For instance, *Babesia divergens* is detected in red deer (*Cervus elaphus*) with relevant frequency, similar to *B. capreoli* in roe deer (*Capreolus capreolus*) (4–6). In contrast, red fox (*Vulpes vulpes*) is believed to act as a reservoir for some *Babesia microti-*like species, such as the previously classified *B. vulpes* (7, 8), and, moderately, for *Borrelia burgdorferi sensu lato* (s.l.) (9). The wild boar (*Sus scrofa*), deer species, and mesocarnivores are all believed to contain *Anaplasma phagocytophilum* (10–12). Wild boars have tested positive for certain pathogens such as *B. vulpes* or *B. capreoli* although they are not considered reservoir hosts for these species (13–15). Spotted fever group (SFG) *Rickettsia* and *B. burgdorferi* s.l. are registered as reservoir species for small mammals and birds (16). Deer species are not reservoirs for or commonly infected by *B. microti*-like (17), *B. burgdorferi* s.l. (18, 19), or SFG *Rickettsia* (20). Similarly, tick species might be specialist vectors for selected pathogens; for example, *Ixodes ricinus* is considered competent for multiple pathogens, while *Haemaphysalis punctata* is the main vector of some SFG *Rickettsia* (16, 21).

In the study of wildlife populations, camera traps (CTs) have been recognized to provide high-quality data for characterizing wildlife communities (22). This can be of utmost use in tick-host interaction studies (23, 24). Nevertheless, in the European context, very few studies have linked CT-derived wildlife data to predict the presence of tick-borne pathogens. Among these, Takumi et al. (25, 26) have employed camera trap data to study the correlation between vertebrate host availability and density of tick-borne pathogens, including *Borrelia* spp. and *A. phagocytophilum*, showing a positive correlation with bank voles and wild ungulates, respectively.

The objective of this study is to assess how effectively camera trap data, specifically head counts of wildlife, can predict the presence of tick-borne pathogens in environmental ticks. This analysis is centered on species readily monitored by such tools, which are also relevant by indirectly influencing tick-borne disease dynamics.

## 2 Materials and methods

### 2.1 Sampling area and study design

**Figure 1:**
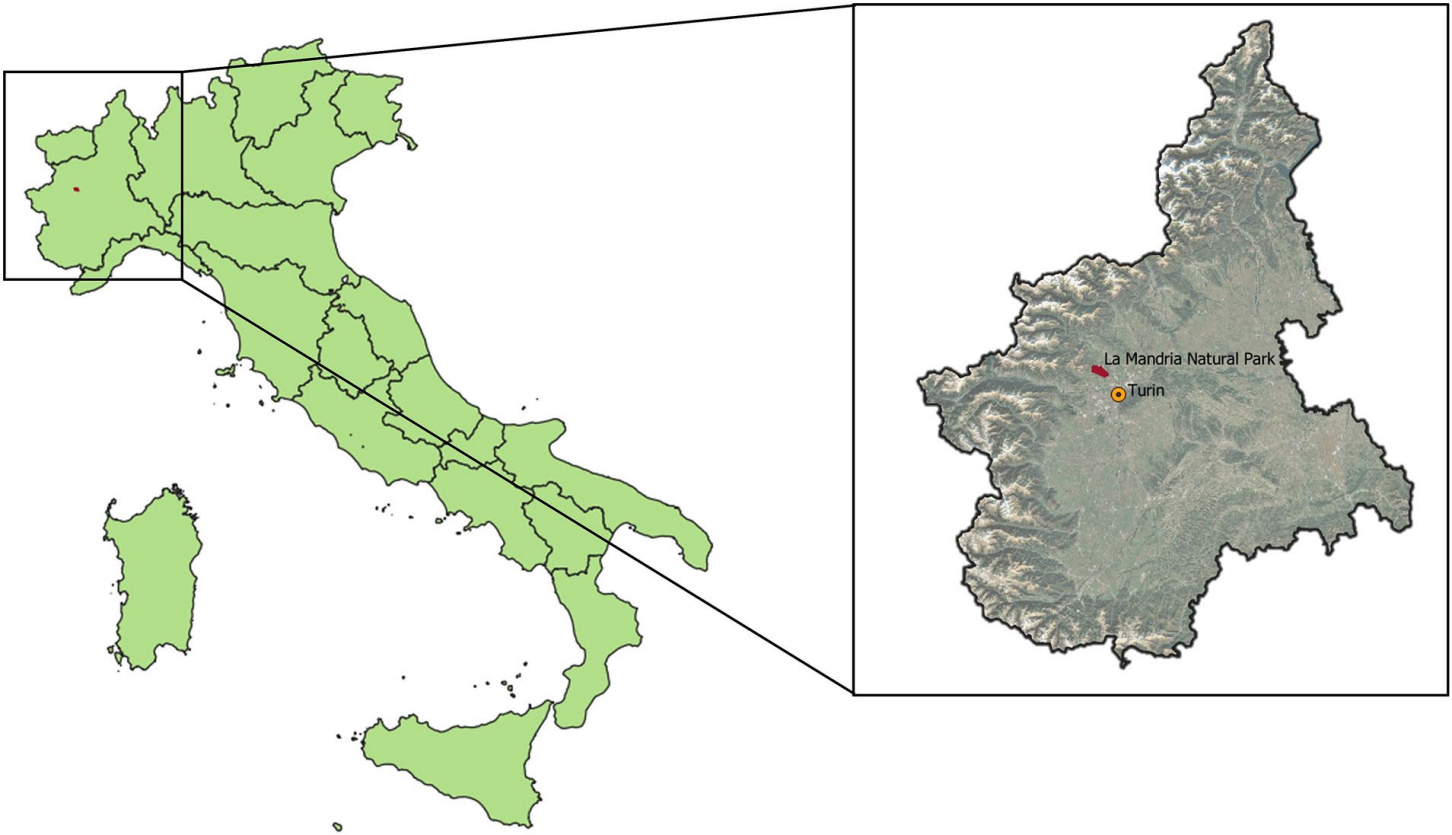
Location of La Mandria Natural Park in the Piedmont region (Italy), with reference to the regional capital, Turin.

The park (45° 8′ 45″, 7° 36′ 2″, Figure 1) spanning 6,571 hectares at an average elevation of 386 m above sea level, with an elevation gradient of 269 m, is characterized by a temperate lowland climate and is enclosed by fencing (27). The park predominantly features deciduous forests and grasslands, which are managed as hay meadows. The park attracts approximately 2,000 visitors daily and maintains consistent wildlife management throughout the year. While a few horse farms and cultivated plots are situated within the park, access by other domestic animals is restricted (including pets). Data on the density of wild ungulates within the park have been recently established via camera trapping by the European Wildlife Observatory (27).

The camera trap images analyzed in this study originated from a separate field study conducted by Ferroglio et al. (23). This study utilized 14 sampling points, evenly distributed between open (hay meadows) and closed (deciduous forests) habitats. Camera traps at each sampling point were operated continuously for a year, from August 2020 to August 2021; for detailed information on the deployment of these traps, refer to Ferroglio et al. (23). Alongside the camera trapping, ticks were systematically collected every 2 weeks throughout the entire study period using dragging transects. As described by Ferroglio et al. (23), we implemented a 1 m^2^ cloth, dragged to cover a 10 m^2^ surface in front of the camera trap and a 26 m circle around it. The cloth was repeatedly checked to collect ticks, which were stored in 70% EtOH for further identification (time of storage: 1 to 2 months).

### 2.2 Tick-borne pathogen detection and prevalence estimation

Alongside fieldwork, ticks were identified using dichotomous keys (28–30), washed to remove any EtOH residual, which would inhibit the polymerase chain reactions, and stored at −20°C for further analysis (performed after the end of fieldwork). To optimize the effort, the genomic DNA was extracted from ticks grouped into uniform pools based on specific criteria: dragging transect, sampling point, repetition, species, developmental stage, and sex for adult ticks. The extraction was performed on the entire body of the ticks in the pool, using a blackPREP Tick DNA/RNA kit (Biosense, Italy) according to the manufacturer’s instructions. Subsequent PCR tests were conducted on these samples to detect tick-borne pathogens, namely *B. divergens*/*capreoli*, *B. microti*-like, *Theileria capreoli*, *A. phagocytophilum*, *B. burgdorferi* s.l., and SFG *Rickettsia*. Table 1 illustrates the references and targeted genes of the primers implemented to detect pathogens’ DNA, while specific protocols are detailed in Supplementary Table S1. Our PCR protocol did not differentiate *B. capreoli* from *B. divergens*, as it did not target the specific region containing single-nucleotide polymorphisms which is typically used to differentiate the two species (31). However, given the specific features of the study area (the absence of domestic ruminants and the surrounding fence) and the documented extensive circulation of *B. capreoli* (6), we considered positive samples to represent *B. capreoli* alone and included them in the statistical analysis. Originally designed to detect *B. microti*, primers from Persing et al. (32) encompass the whole *B. microti*-like group, including vulpes-like and Munich-like clades (8), which are of interest both from a zoonotic point of view and for the wildlife species targeted in the present study, specifically the red fox. Primers for *T. capreoli* were designed in the current study to be species-specific. All primers for Piroplasmid species were intended to avoid the interference of co-infections with other protozoan microorganisms. Similarly, when targeting Anaplasmataceae, we selected specific primers for *A. phagocytophilum*, the species with the highest public health relevance, to avoid the interference of symbiotic bacteria such as *Candidatus* Midichloria spp. On the other hand, primers targeting *B. burgdorferi* s.l. and SFG *Rickettsia* encompass the whole group of microorganisms (e.g., *B. afzelii* and *B. lusitaniae* in the first case, and *R. monacensis* and *R. conorii* in the second), which are all relevant for public health as zoonotic pathogens and are not commonly hosted by the wild species targeted in the study (16). All PCR tests included a confirmed positive control for the target pathogen and a no-template negative control. All standard measures were taken to minimize the risk of contamination. Amplicons were analyzed by agarose gel electrophoresis (2%) and visualized by staining with GelRed Nucleic Acid Gel Stain (VWR International Milano, Italy).

**Table 1:** Primers implemented in the study, with gene-targeted, primer names and publication reference.

Pathogen	Primer names (5′–3′)	References
*B. divergens*	Gene: *18s* (forward and reverse from the paper)	(31)
*B. microti/microti-*like	Gene: *18s* (Bab1 and Bab4)	(32)
*A. phagocytophilum*	Gene: *groEL* [EphplgroEL(569)F and EphplgroEL(1193)R]	(65)
*B. burgdorferi* s.l.	Gene: spacer region between *5S* and *23S rRNA* genes (23SN1 and 23SC1)	(66)
SFG *Rickettsia*	Gene: surface protein *rOmpA* (190-70 and 90-701)	(67)
*T. capreoli*	Gene: *18s* (TcapreoliF and TcapreoliR)	This study. Reference sequence: AY726011.1

For each pool and pathogen, we recorded the binary outcome of the PCR (positive-negative) and estimated the pathogen prevalence within each pool using the package PoolTestR (33) for RStudio (34). This package provides Bayesian estimates of prevalence along with 95% credibility intervals (Cr.I.), based on the number of ticks in each pool and the test outcome (33). Differences in prevalence among tick species and among developmental stages were explored through the chi-squared test or Fisher’s test, depending on the distribution matrix (35).

### 2.3 Tick-borne pathogen models

For each repetition and sampling point, we computed the total head count of individuals passing by the camera trap. Each instance of an animal exiting and re-entering the camera’s field view was treated as a new individual, as individual recognition was not possible. All age classes were included in the analysis. We extracted data for red deer, roe deer, fallow deer (*Dama dama*), wild boar, red fox, European badger (*Meles meles*), pine marten (*Martes martes*), and beech marten (*Martes foina*), as CT deployment was not sensitive for distinguishing animal species of smaller size, such as rodents and birds, or for detecting their presence in the whole field of view of the camera trap. We grouped data for mesocarnivores and wild ruminants.

Our database considered, as the response variable, the binary outcome of the PCR test (positive-negative). Explanatory variables involved were as follows:

- Sampling season and point.
- Wildlife head counts (as number of passages) for wild boar, mesocarnivores, and wild ruminants.
- The number of ticks per pool, as the more ticks are tested together, the higher the probability of getting a positive PCR test, i.e. detect an infection.

Vegetation and environmental parameters were not included in the analysis for several reasons. First, the sampling points were uniformly distributed across vegetation types, ensuring homogeneity in coverage. Second, the habitat within the park exhibited overall uniformity due to its limited spatial extent and minimal elevation gradient. Finally, the influence of vegetation on the presence of tick-borne pathogens was likely indirect, as vegetation primarily affects reservoir host distribution or vector abundance rather than directly influencing the pathogen presence.

We implemented a General Linear Mixed Model with a binomial family using the *lme4* package in R studio (36). We considered the sampling point as the random variable, and the variance was weighted on the season in which ticks were collected, to account for seasonality in tick and host population.

To test how well the model performed and, consequently, how useful camera trap data could be to predict the presence of tick-borne pathogens, we evaluated three parameters: (i) conditional and marginal *R*^2^, to test how much the model was satisfactory in explaining the variance; (ii) accuracy; and (iii) AUC (area under curve, for which threshold interpretation was presented by studies such as Çorbacıoğlu et al. (37)), tested by splitting the database into train (70% of data) and test (30% of data) datasets.

We modeled (i) all tick pools, regardless of species and developmental stages (global model), and (ii) *I. ricinus* nymph pools, as particularly relevant in terms of zoonotic risk (*I. ricinus* nymph model) (38, 39). We ultimately created models only for the four most prevalent pathogens (*B. capreoli*, *B. microti-like*, *B. burgdorferi* s.l., and SFG *Rickettsia*).

## 3 Results

### 3.1 Tick-borne pathogen detection and prevalence estimation

We analyzed a total of 2,537 ticks divided into 413 pools, including 282 pools of *I. ricinus*, of which 112 (424 individuals) were identified in the nymphal stage (23). The two most prevalent pathogens were *B. microti*-like (Bayesian prevalence 13.12% with Cr.I. 11.05–15.28%) and SFG *Rickettsia* (10.79%, Cr.I. 9.14–12.64%), followed by *B. capreoli* (5.47%, Cr.I. 4.2–6.78%) and *B. burgdorferi* s.l. (2.57%, Cr.I. 1.83–3.45%). Finally, the prevalence was recorded below 1% for *T. capreoli* and *A. phagocytophilum*, respectively (0.23%, Cr.I. 0.02–0.71, and 0.34%, Cr.I. 0.14–0.62%).

*Ixodes ricinus* and *H. punctata* recorded at least one positive pool for each pathogen (Table 2). *B. capreoli*, *B. microti*-like, and SFG *Rickettsia* were found in samples from every tick species tested, including *D. reticulatus*, *H. concinna*, *I. hexagonus*, and the *R. sanguineus* complex [*R. sanguineus sensu stricto*, *R. pusillus*, and *R. turanicus* according to the keys implemented in this study (28, 30)]. Additionally, *T. capreoli* was detected in *H. concinna*, *H. punctata*, and *I. hexagonus*, while *B. burgdorferi* s.l. was identified in *H. concinna* and *H. punctata*. According to the chi-squared test and Fisher’s test, the prevalence rates of only *B. burgdorferi* s.l. were significantly different (*p* < 0.05) among developmental stages and those of *B. microti*-like alone did significantly vary (*p* < 0.05) among the tick species.

**Table 2:** Bayesian estimated the prevalence of pathogens for each tick species and their developmental stages, with 95% Cr.I. within parentheses.

Tick species	*B. capreoli*	*B. microti*-like	*B. burgdorferi* s.l.	SFG *Rickettsia*	*A. phagocytophilum*	*T. capreoli*
*I. ricinus* [393]						
Larva [154]	3.5% (2.3–5%) [29]	6.5% (4.9–8.2%) [54]	0.4% (0.1–0.8%) [4]	7.5% (5.8–9.6%) [60]	0.2% (0–0.6%) [2]	0.5% (0.2–1%) [5]
Nymph [184]	7.5% (5–10.7%) [26]	30.9% (24.3–37.8%) [68]	8.2% (5.5–11.5%) [25]	31.2% (24–39.1%) [60]	0.7% (0.1–1.7%) [2]	0.8% (0.2–2%) [3]
Adult [55]	23.1% (12.1–36.7%) [9]	53.5% (37.4–69.5%) [20]	22.9% (11–37.1%) [9]	38.4% (24.1–52.7%) [15]	6% (1.1–14%) [2]	[0]
*I. hexagonus* [4]						
Nymph [4]	24.4% (2.5–60.1%) [1]	42.1% (8.7–77.2%) [2]	[0]	24.9% (3.1–62.8%) [1]	[0]	[0]
*H. concinna* [27]						
Larva [9]	1.1% (0.2–3%) [2]	1.1% (0.2–2.9%) [2]	0.7% (0–2.1%) [1]	2.1% (0.7–4.4%) [4]	[0]	[0]
Nymph [15]	12.8% (4.3–25.8%) [4]	4.2% (0.3–13%) [1]	4.3% (0.4–13.1%) [1]	19.3% (8.2–33.8%) [6]	[0]	10.2% (2.4–22.2%) [3]
Adult [3]	49.8% (5.6–94.3%) [1]	49.7% (5–95%) [1]	50.4% (7.2–93.8%) [1]	[0]	[0]	[0]
*H. punctata* [43]						
Larva [33]	1.6% (0.7–2.7%) [9]	0.5% (0.1–1.2%) [3]	0.2% (0–1%) [4]	3.9% (2.2–6.2%) [16]	[0]	0.2% (0–0.7%) [1]
Nymph [10]	16.5% (3–36.9%) [2]	16.6% (2.7–38.7%) [2]	9.6% (0.8–29.1%) [1]	29.5% (10.4–53.7%) [4]	9.8% (0.7–27.8%) [1]	[0]
*R. sanguineus* complex [11]						
Larva [8]	9.7% (1.9–23.6%) [2]	14.3% (3.6–29.8%) [3]	[0]	14.5% (3.8–31.8%) [3]	[0]	[0]
Nymph [3]	50.3% (6.2–94.1%) [1]	49.6% (5–93.8%) [1]	[0]	50% (5.4–95.2%) [1]	[0]	[0]
*D. reticulatus* [2]						
Larva [1]	35.1% (2.6–80.6%) [1]	[0]	[0]	[0]	[0]	[0]
Adult [1]	[0]	[0]	[0]	36.5% (3.5–82%) [1]	[0]	[0]

The number of pools per group (left column) and the number of positive pools (other columns) are given in squared brackets.

**Figure 2:**
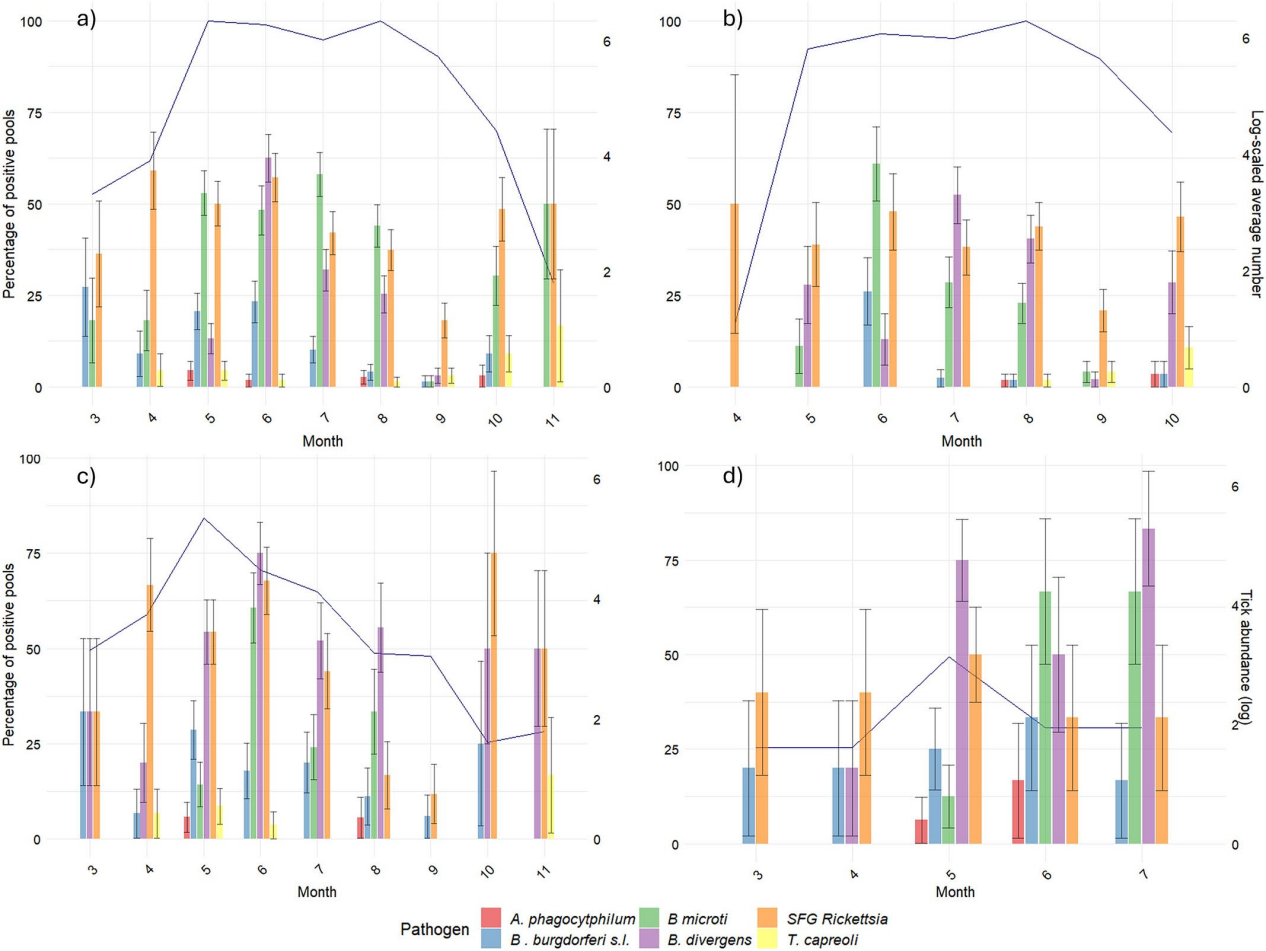
Pathogen positivity across the months per developmental stage. For each of the targeted pathogens, the percentage of positive pools over the total number of collected pools in each month is shown on the primary y-axis. Winter months (December, January, and February) were excluded due to a lack of tick activity. The blue line (secondary *y*-axis) represents ticks’ abundance (log-scaled average of number of ticks collected in each sampling point). **(A)** Represents the total number of ticks. **(B)** Represents the number of larvae. **(C)** Represents the number of nymphs. **(D)** Represents the number of adults.

While the percentages of the positive pools of SFG *Rickettsia* and *A. phagocytophilum* were persistent throughout the study period, we observed a peak during the summer months for *B. burgdorferi* s.l. and the two *Babesia* species (Figure 2). However, some differences may be spotted while decomposing the trend according to developmental stages. For *B. burgdorferi*, positive larvae peaked in June, while among the other stages, positive pools were more uniformly distributed in warmer months (May–September). For *B. microti*-like, they peaked in all stages in June and July. Finally, for *B. divergens*, the peak in nymphs and adults occurred earlier in the year (from May to July) than in larvae (July). *Theileria capreoli*, which was almost only recorded in larvae, showed higher positivity later in the year.

### 3.2 Tick-borne pathogen models

Based on the best performance of the models, the number of ticks in the pool and the wild boar and mesocarnivore head counts were included in all models (Table 3), while deer species head counts were included in the models for *B. capreoli*, SFG *Rickettsia* and in the global model of *B. burgdorferi* s.l.

**Table 3:** Variables included in each model.

Model	Pathogen	Wild boar	Deer species	Mesocarnivores	Tick pool
Global model	*B. capreoli*	✓	✓	✓	✓
	*B. microti*-like	✓		✓	✓
	*B. burgdorferi* s.l.	✓	✓	✓	✓
	SFG *Rickettsia*	✓	✓	✓	✓
*I. ricinus* nymph model	*B. capreoli*	✓	✓	✓	✓
	*B. microti-*like	✓		✓	✓
	*B. burgdorferi* s.l.	✓		✓	✓
	SFG *Rickettsia*	✓	✓	✓	✓

Considered explanatory variables are wild boar, deer species, mesocarnivores head counts, and the number of ticks in the pool. Both the global model and the model for *I. ricinus* nymphs are described.

**Figure 3:**
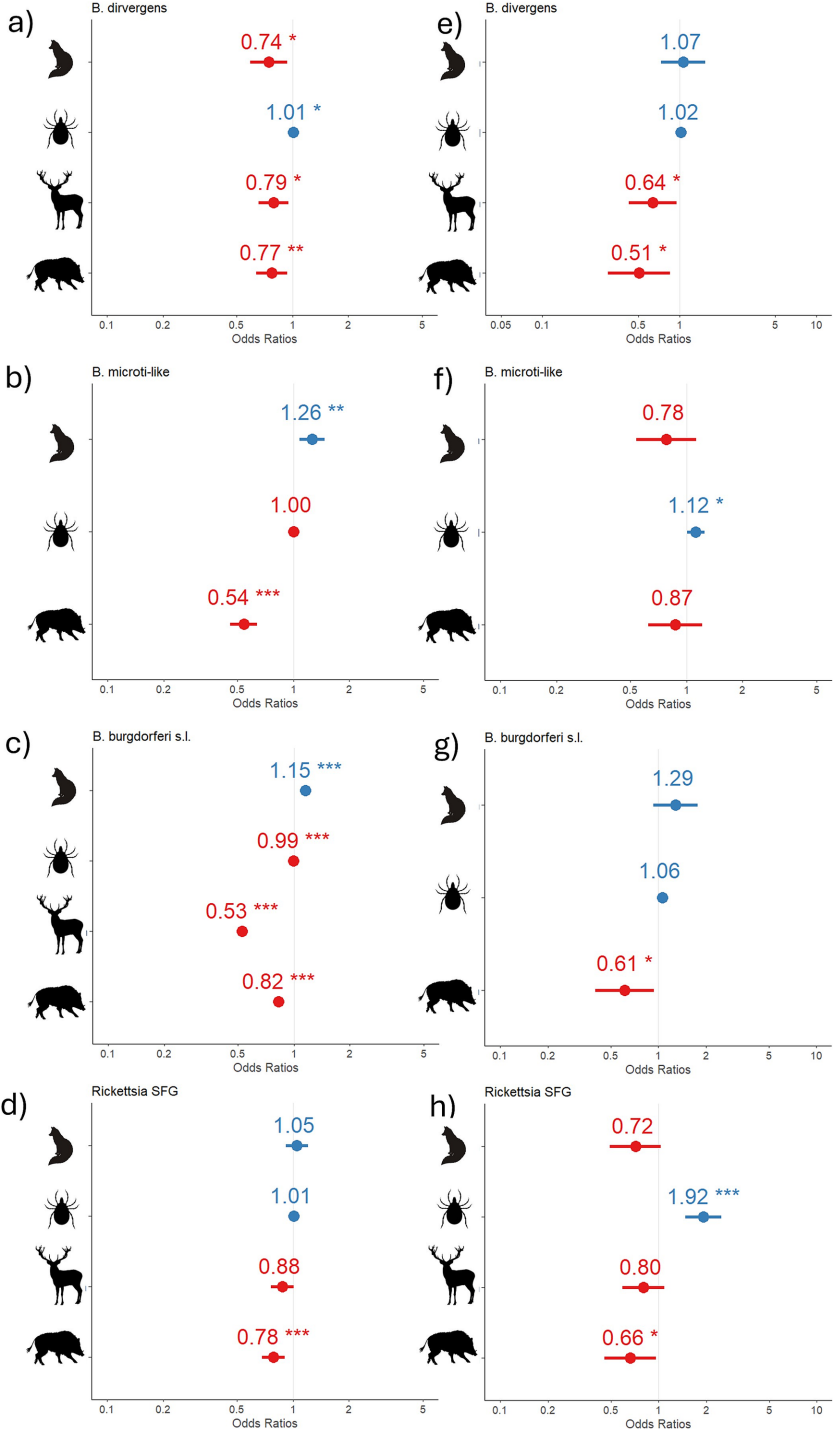
Model result coefficients. Coefficients (blue, positive; red, negative) of the models for each pathogen in the global model **(A–D)** and in the *I. ricinus* nymph model **(E–H)**. Asterisks indicate statistical significance. Silhouettes on the *x*-axis represent mesocarnivores head count (fox), total number of ticks in the pool (tick), wild ruminants head count (red deer), and wild boar head count (wild boar).

Overall, the coefficients indicated a similar effect (either dilutive or additive) of the same host species across both the global model and the *I. ricinus* nymph model, despite statistical significance (Figure 3). Wild ruminants and wild boar exhibited a dilutive effect (negative coefficient) on the prevalence of all assessed pathogens. In contrast, the influence of mesocarnivores varied between the global model and the *I. ricinus* nymph model. Overall, mesocarnivores demonstrated a statistically significant dilutive effect for *B. divergens*, while showing a statistically significant additive effect for *B. burgdorferi* s.l. and *B. microti*-like. Furthermore, the total number of ticks in the pool had a positive association with the presence of pathogens within the pool, except in the global model of *B. burgdorferi* s.l., where a detractive impact was detected.

The model performance, evaluated using *R*^2^, accuracy, and AUC, was the highest for the models focused on *I. ricinus* nymphs, apart from those predicting *B. burgdorferi* s.l. Notably, SFG *Rickettsia* exhibited the lowest accuracy and AUC, but the highest *R*^2^ among the models analyzed. The model for *B. capreoli* demonstrated the highest accuracy, while the *B. burgdorferi* model achieved the highest AUC, exceeding 0.7, marking it as the only model to surpass this threshold of good predictivity (Table 4).

**Table 4:** *R*^2^ values, accuracy, and AUC for each model.

	Global model	*I. ricinus* nymph model
*B. divergens*		
Marginal *R*^2^/conditional *R*^2^	0.070/0.204	0.188/0.227
Accuracy	0.74	0.9
AUC	0.65	0.65
*B. microti-*like		
Marginal *R*^2^/conditional *R*^2^	0.098/0.171	0.137/0.485
Accuracy	0.6	0.69
AUC	0.62	0.67
*B. burgdorferi* s.l.		
Marginal *R*^2^/conditional *R*^2^	0.098/0.388	0.118/0.429
Accuracy	0.91	0.86
AUC	0.66	0.74
SFG *Rickettsia*		
Marginal *R*^2^/conditional *R*^2^	0.031/0.049	0.862/0.876
Accuracy	0.56	0.57
AUC	0.59	0.63

## 4 Discussion

Our models showed that the wildlife presence affects the prevalence of tick-borne pathogens, and consequently, camera trap data can be useful for predicting their risk in the environment.

The prevalence of *Rickettsia*, *A. phagocytophilum*, *Babesia* spp., and *B. burgdorferi* s.l. in this study was consistent with that reported in studies on *I. ricinus* ticks collected from dogs (40), humans (41), and wildlife (6, 42) in northwestern Italy and in previous studies in the same study area (43). The prevalence detected for *T. capreoli* was consistent with that reported in wild deer species in Spain (44, 45). Temporal fluctuations in the pathogen presence did follow the seasonal peaks of the developmental stages in which they were more often detected (23), which is also in accordance with the pool size variable in the model showing an additive effect on the presence of the pathogens. Pathogens capable of transovarial transmission, such as *B. divergens* and SFG *Rickettsia* (46, 47), exhibit a more evenly distributed positivity rate throughout the months of tick activity. In contrast, *B. microti*-like, which lacks transovarial transmission (48), shows a peak in positivity rates concentrated in June. Despite its transovarial transmission, *B. burgdorferi* s.l., in accordance with results obtained by Szekeres et al. (49) in Germany, was predominantly found in nymphal pools and its temporal fluctuations varied accordingly with this developmental stage, with a peak in June. This concurrence was also highlighted by Hartemink et al. in the Netherlands (50).

A limitation of the minimum infection rate (MIR) estimation is that it is derived from the number of ticks within a pooled sample (MIR = 1/*n*° ticks). Consequently, as the number of ticks tested together increases, the denominator rises, leading to a lower calculated prevalence. Although the Bayesian approach partially mitigates this issue, it does not eliminate the bias introduced by the pool size: for instance, a study on tick-borne zoonoses in the same study area testing individual ticks detected higher prevalence for SFG *Rickettsia* and *B. burgdorferi* s.l. (43). This is particularly relevant for larval pools, which may encompass a substantial number of ticks, potentially resulting in an underestimation of the true prevalence. This approach is however more precise than other traditionally implemented methods and still allows comparison among tick species or different locations, especially in situations where single tick testing would be poorly effort-effective (51). In addition to these considerations, it is noteworthy that an increased number of ticks positively affects the probability of a positive outcome of a biomolecular test, remarking how tick hotspots may represent a major risk for tick-borne disease transmission. The sole exception was the effect observed for *B. burgdorferi* s.l. in the global model, where this effect was highlighted as detractive. The scarcity of positive detection during periods of higher larval abundance may have influenced this outcome.

In some cases, pathogens found in tick species were not recognized as competent vectors: for instance, *Babesia* spp. were found in all species, although only *I. ricinus* has been demonstrated to be capable of transmission back to the vertebrate host (16, 21). Even though little is known about the actual competence of less common tick species, it is important to clarify that the presence of pathogen DNA in the vector is not proof of the transmission capability, and it just mirrors a pathogen’s circulation in the study area. This can explain the absence of a statistically significant prevalence difference among tick species for most pathogens. According to the literature, *B. burgdorferi* was detected in *H. punctata* (52), *B. microti* in *D. reticulatus* (53), and *B. bigemina* in *H. punctata* (54), thus confirming our findings that pathogen infection and transmission do not necessarily coincide.

While the wild ungulate species targeted by our camera trap data collection are likely not reservoirs for the four modeled pathogens (13, 15, 17, 18, 20), they serve as maintenance hosts for the tick population (23, 55, 56). In light of informing management actions aimed at reducing the number of ticks, we chose them as relevant variables to model. Despite *B. capreoli* having been reported in red deer (6), there are several cases where only roe deer was found positive for this pathogen (57–59). Additionally, *B. capreoli* was not reported in red deer in areas where roe deer was absent (4), suggesting a limited role of this species as a maintenance host. This probably explains our finding of a dilutive effect of deer species altogether, as roe deer density in the park was 1.90 ± 0.97 ind/km^2^, much lower than that of other species (27), and the ratio between roe and red deer head counts in our study was 171:1,212 and that between roe and fallow deer was 171:854. In Europe, *B. microti*-like species include *B. vulpes* and the *B. microti* Munich strain (8). Red fox, the mesocarnivore species mainly recorded in our study, may harbor *B. vulpes*, which is a possible reason for the additive effect of its presence on *B. microti*-like in environmental ticks. Additionally, the presence of red fox may be linked to its micromammal prey, which can be an underlying factor for the positive coefficient on *B. burgdorferi* s.l. and on *B. microti-*like. Indeed, both pathogens have micromammals, such as ground-dwelling rodents and shrews, as major reservoirs (8, 16). Including small mammals in such types of studies will be a fundamental step forward in understanding the ecology of these tick-borne pathogens, as performed by Takumi et al. (25).

In this study, and for the reasons outlined, we did not incorporate environmental variables into the analysis. While temperature and humidity significantly influence tick activity and abundance (60), and consequently the prevalence of tick-borne pathogens by increasing tick population density and contact rates between hosts, habitat characteristics primarily affect host presence, density, and temporal occupancy (23, 61, 62). These, in turn, indirectly influence tick abundance and pathogen presence when hosts serve as competent reservoirs (23, 24, 63, 64). Although parameters such as vegetation types or habitat fragmentation indices could be included in the analysis as proxies for the presence of other host species, they would lack the precision of data obtained directly from camera traps. Our results highlighted how camera trap data represent a valid tool to predict the presence of tick-borne pathogens and, consequently, draw insights about the zoonotic risk and further control strategies. The model performance in predicting the presence of pathogens is improved by decomposing the response variable, indicating that pathogen-host association may vary depending on the developmental stage and species of the vector. More accurate predictions and new insights on the pathogen-host interaction would benefit from *ad hoc* models targeting the single species and developmental stages. This approach was not possible in the current study due to limited numbers of other tick species.

## 5 Conclusion

Our study verified the presence of tick-borne pathogens in a fenced natural park, a site frequented by many visitors engaging in various outdoor activities. While rodents are known to be primary reservoirs and maintenance hosts for several of these pathogens, our research concentrated on species that indirectly influence pathogen transmission. These species (wild ungulates in particular and red fox to a lesser degree) are more readily observed and managed, particularly through camera trapping and hunting. Our findings demonstrate a clear connection between pathogen prevalence and these species, underscoring the value of camera trap data in providing detailed insights into wildlife populations for studies in disease ecology.

## Data Availability

The raw data supporting the conclusions of this article will be made available by the authors upon request.

## Supplementary material

SUPPLEMENTARY TABLE 1 Protocols and primers implemented in the study.
