# Supplementary material for: Assessing zoonotic risk in a fenced natural park in northwestern Italy: integrating camera traps for a vector-host approach to investigate tick-borne pathogens
Source: Front Vet Sci. 2025 Mar 3;12:1536260. doi: 10.3389/fvets.2025.1536260 (PMC11911494; doi:10.3389/fvets.2025.1536260)
Supplement: SUPPLEMENTARY TABLE 1 — Protocols and primers implemented in the study. [file Table_1.DOCX]

Supplementary Material

**Supplementary Table 1.** Protocols and primers implemented in the study

| Target pathogen | Primers (5’ – 3’) | Thermic profile | Reaction mixture  (25 ul total volume) | Original reference |
| --- | --- | --- | --- | --- |
| *B. divergens* | Gene: *18s*  Forward: GTTTCTGMCCCATCAGCTTGAC  Reverse: CAATATTAACACCACGCAAAAATTC | 40 cycles:  94°C - 30”  55°C - 20”  72°C - 30” | PCR Master Mix: 1X  Primers: 10 pmol  DNA: 1 μl | (1) |
| *B. microti* | Gene: *18s*  Bab1:  CTTAGTATAAGCTTTTATACAGC  Bab4: ATAGGTCAGAAACTTGAATGATACA | 40 cycles:  94°C - 30”  51°C - 20”  72°C - 30” | PCR Master Mix: 1X  Primers: 10 pmol  DNA: 1 μl | (2) |
| *A. phagocytophilum* | Gene: *groEL*  EphplgroEL(569)F: ATGGTATGCAGTTTGATCGC  EphplgroEL(1193)R:  TCTACTCTGTCTTTGCGTTC | 40 cycles:  94°C - 30”  55°C - 30”  72°C - 45” | HotStarTaq DNA Polymerase: 2.5 U  Primers: 25 pmol  DNA: 2.5 μl  dNTPs mix: 0.2 mM PCR buffer: 1X | (3) |
| *B. burgdorferi* s.l. | Gene: spacer region between *5S* and *23S rRNA* genes  23SN1: ACCATAGACTCTTATTACTTTGAC  23SC1:  TAAGCTGACTAATACTAATTACCC | Touch down:  94°C - 20”  60°C - 30” (-1°C every 2 cycles)  72°C - 30”  25 cycles:  94°C - 20”  50°C - 30”  72°C - 30” | HotStarTaq DNA Polymerase: 2.5 U  Primers: 20 pmol  DNA: 2.5 μl  dNTPs mix: 0.2 mM  PCR buffer: 1X | (4) |
| SFG *Rickettsia* | Gene: surface protein *rOmpA*  190-70:  ATGGCGAATATTTCTCCAAAA  90-701:  GTTCCGTTAATGGCAGCATCT | 40 cycles:  94°C - 30”  55°C - 30”  72°C - 1’ | HotStarTaq DNA Polymerase: 2.5 U  Primers: 7 pmol  DNA: 5 μl  dNTPs mix: 0.2 mM  PCR buffer: 1X | (5) |
| *T. capreoli* | Gene: *18s*  Forward: TGTGGCTTATTTCGGTTATAAAAT  Reverse: AAAAGCTTATTCCCGTACCCTA | 40 cycles:  94°C - 30”  55°C - 30”  72°C - 1’ | PCR Master Mix: 1X  Primers: 25 pmol  DNA: 1 μl | This paper. Reference sequence accession number: AY726011.1 |

1. Hilpertshauser H, Deplazes P, Schnyder M, Gern L, Mathis A. Babesia spp. identified by PCR in ticks collected from domestic and wild ruminants in southern Switzerland. Appl Environ Microbiol. 2006;72(10):6503–7.

2. Persing DH, Mathiesen D, Marshall WF, Telford SR, Spielman A, Thomford JW, et al. Detection of Babesia microti by polymerase chain reaction. J Clin Microbiol. 1992;30(8):2097–103.

3. Alberti A, Addis MF, Sparagano O, Zobba R, Chessa B, Cubeddu T, et al. Anaplasma phagocytophilum, Sardinia, Italy. Emerg Infect Dis. 2005;11(8):1322.

4. Rijpkema SG, Molkenboer MJ, Schouls LM, Jongejan F, Schellekens JF. Simultaneous detection and genotyping of three genomic groups of Borrelia burgdorferi sensu lato in Dutch Ixodes ricinus ticks by characterization of the amplified intergenic spacer region between 5S and 23S rRNA genes. J Clin Microbiol. 1995;33(12):3091–5.

5. Roux V, Fournier P-E, Raoult D. Differentiation of spotted fever group rickettsiae by sequencing and analysis of restriction fragment length polymorphism of PCR-amplified DNA of the gene encoding the protein rOmpA. J Clin Microbiol. 1996;34(9):2058–65.
